# Epidemic Cerebro-Spinal Meningitis

**Published:** 1873-02

**Authors:** Charles Forbes

**Affiliations:** Rochester, N. Y.


### ART. III.—*Epidemic Cerebro-Spinal Meningitis.* By CHARLES FORBES, M. D., Rochester, N. Y.

Read before the Central New York Medical Association.

*Synonyms.* Cerebro-spinal fever. Malignant purpuric fever. Spotted fever. Also been called typhoid meningitis and malignant meningitis.

*Definition.*—Dr. Aitken defines the disease to be a malignant epidemic fever of an acute, specific character, of sudden invasion, attended by painful contraction of the muscles of the neck, and retraction of the head. In certain epidemics it has been frequently accompanied by a profuse puerperic eruption, and occasionally by secondary effusion into certain joints. Lesions of the brain, the spinal chord, and their membranes, are found on dissection. The course of the disease is rapid and very fatal, attended with great prostration of the powers of life, severe headache, and pain along the spine.

*History.*—It is believed that this disease prevailed epidemically in Europe at different periods of the 14th, 16th and 17th centuries. In the United States it was first seen in the epidemic form at Medfield, Mass., in 1806, and from that time until 1816 it was constantly epidemic throughout this country, particularly in the New England States. From 1816 to 1860 casual epidemics, with sporadic cases here and there, seemed to have occurred in several of the States, particularly the Southern and Western, the Middle and Eastern having been comparatively exempt. In 1863-'64-'65 it prevailed as an epidemic of wide range and with great mortality in West Prussia. About the same time a very fatal disease devastated parts of Russia, which is believed to have been cerebro-spinal meningitis.

In March, 1866, it prevailed as an epidemic in Dublin, and reached its height during the following year. It spread to other towns in Ireland, and there were some cases in England.

It prevailed to a considerable extent amongst the troops of the U. S. Army during the late war, and also in the confederate army. It was first seen during the winters of 1861 and '62 in the army of the Potomac.

*Morbid Anatomy.*—The morbid appearances found in the cerebro-spinal axis and its membranes are: When death has happened within two or three days after the invasion, opalescence of the upper surface of the cerebrum, seemingly in the subarachnoid fluid; an abundant vascularity of the membranes of the brain, chiefly of the pia-mater; a large increase of serum in the subarachnoid space and in the ventricles, clear or turbid and mixed with flocculi of lymph, and as often as otherwise, even in cases of the briefest duration, an abundant exudation of thick, yellowish, apparently semi-organized lymph at the base of the brain and the medulla oblongata.

The membranes at this stage are *sometimes* remarkably dry, without injection; or they may be adherent to the surface of the brain or among themselves. The cerebro-spinal fluid is much increased, and of a yellowish and milky hue; the spinal meninges congested like those of the brain; and the chord has been found softened.

In all epidemics of this disease cases have occurred in which no appreciable changes have been found in the cerebro-spinal membranes.

Thus during the prevalence of the disease in the army there appears to have been two classes; in the first, the autopsy disclosed grave anatomical lesions of the cerebro-spinal axis, accumulations of serum, sero-pus, pus or tough yellow lymph, especially in the ventricles about the base of the brain and in the upper part of the spinal canal; in the second class of cases no perceptible anatomical lesion in the cerebro-spinal axis was observable. Both anatomical conditions appear to have been found indifferently, in protracted cases as well as in those which proved suddenly fatal.

*Blood.*—The blood is usually very dark colored and fluid, even in the briefest cases. Dr. Stillé states that in some instances firm, fibrinous clots have been found in the heart after death.

*The coincident lesions* are: congestion and œdema of the lungs, pleural, pericardial, and articular sero-purulent effusions, and occasionally enlargement of the glands of Brunner and Pyer, without ulceration.

*Symptoms.*—As a rule the invasion is sudden, without warning indisposition, except, perhaps, general weariness, or aching of the whole body, or shivering, which may amount to a sharp chill, and is often the initial symptom. In some cases weariness and a sense of general uneasiness are felt for a day or two before the onset of the acute symptoms.

The onset of the disorder is almost constantly marked by severe headache, usually occipital, sometimes frontal; the patient speaks of it as acute, excruciating, and unbearable. Dizziness may be complained of; pains in the nape of the neck, limbs, calves of the legs, and joints, particularly the knee-joints. Stiffness of the jaw and neck are felt. Sore throat in some epidemics has been a common initial symptom. There are often soreness and tenderness at the back of the neck and along the spine. Nausea and vomiting may happen at the outset. Generally there is great weakness from the beginning.

*Delrium* may set in soon; it is generally not violent, but wandering or talkative, or muttering; in other cases there may be initial stupor, or even coma; again, there are cases in which the intellect may be unimpaired throughout the attack.

*Eruption.*—From a few hours to one or more days the eruption is seen upon the neck, abdomen, back, arms, legs, and sometimes the face. It is made up of distinct, dark-red or purple blood spots of the size of a pin's head to that of a dime ; they are not raised, and do not fade on pressure. Sometimes they are only dark mottled scattered here and there over the skin. The eruption is not constant, and in some cases it has appeared only after death.

Cutaneous hyper-æsthesia is a symptom that is soon added, and sometimes to such a degree that, slightly touching or brushing the skin, will bring on reflective muscular contractions.

*Anæsthesia* is very rare, and generally at a late stage.

The muscles of the nape of the neck become rigid and retracted, and the head is thrown back ; this is one of the most constant and persistent symptoms.

The tongue may now become dry, dark-colored, or even black, fissured and swollen, or covered with sores.

The bowels, as a rule, are constipated.

Disorders of the organs of sight and hearing are not constant symptoms ; squinting, and some degree of deafness and a buzzing in the ears, are the most common.

The patient usually lies on the side, at least until towards the last, though there are usually much restlessness and tossing about.

*Death* happens by coma, or by paralysis of the heart and lungs, or possibly by asthenia.

*The duration* of an attack is from a few hours to many weeks. Cases are recorded which have ended fatally in three, four and five hours. More than one-half the deaths are said to happen from the second to the fifth day. In the late Irish epidemic a large proportion of the fatal cases died in from ten to forty-eight hours; in others, death happened at the end of the second and during the course of the third week.

*Convalescence*, taking place from the fifth day to the fourth week, and often later, is tedious, and full health is not regained sometimes for months.

Dr. Clymer is of the opinion that relapses in this disease are frequent.

Dr. Flint states that they have not been observed.

*Mortality.*—Most epidemics and endemics of cerebro-spinal meningitis have been marked by great fatality; in some instances throughout their whole course, in others only for a while after the outbreak. The death rate in the several epidemics between 1838 and 1865 varied between 75 per cent. and 20 per cent.

Dr. Stillé states that ten epidemics in various places between 1838 and 1848 gave an average mortality of 70 per cent., and a like number between 1855 and 1865 gave only about 30 per cent.

*Etiology.*—Of the host of predisposing and exciting causes of this disease which have been catalogued by writers, all have been named in connection with other disorders, endemics and epidemics, and most, or all, have been wanting in some outbreaks of cerebro-spinal meningitis, or in the several localities where it has prevailed, or in individual cases. In this respect nothing constant has been noticed. As regards temperature, we find that the disease has prevailed every month in the year. The outbreaks in this country have been chiefly during the winter and early spring. It has prevailed among all classes and conditions of people. In some epidemics males have suffered most, and in others females have been the victims. This disease appears to be confined to no particular age. Instances are recorded where it has been confined to masses of people living together as in workhouses, prisons, schools, and particularly in barracks.

The mass of testimony is against its being contagious; but cases of apparent communication of the disease from the sick to the well are recorded. There is reason, therefore, to believe that the effective cause of this disease is to be sought for beyond physical and bodily conditions; that it is outside of the degree of heat, moisture, &c., and the constitutional state of the individual. So at the present day we do not know the true cause of the disease, and we only assume that there is a specific poison as the effective cause.

*Nature.*—Of the nature of this disease, but little more appears to be known than there is of its cause. Many have regarded it as a form of simple acute cerebro-spinal meningitis. The objections to this are, that this disease as a primary idiopathic affection occurs very infrequently, and that epidemics or endemics of it are very improbable; and it may be urged that the behavior and the results of treatment of the disease under consideration are not explained upon the supposition of its being a simple meningeal inflammation. The clinical history of epidemic cerebro-spinal meningitis shows that the whole system is implicated from the outset, and it often strikes down its victims without leaving any trace of local damage. Nor is there any constant relation between the severity and duration of the symptoms and the degree and extent of changes in the cerebro-spinal membranes. They are present or absent alike in protracted cases as in those which have proved suddenly fatal.

There are those who believe that epidemic cerebro-spinal mengitis should be classed with the so-called zymotic diseases, and many consider it but a variety of typhus fever. It is urged that there are certain distinguishing marks which should prevent any confusion between the two diseases, the principal of which are, the suddenness of the onset in epidemic cerebro-spinal meningitis, its rapid course, the absence of the mulberry rash of typhus, the early appearance of the hæmic spots, and its non-contagiousness, whilst many of the characteristic symptoms of the continued fever are wanting. It has also been regarded as a form of paludal fever; but Dr. Aitken is of the opinion that there are no sufficient grounds to believe it to be of malarious origin.

*Treatment.*—There is no known antidote to the specific ætic poison, nor can it be expelled by elimination. The indications are to stay, if possible, the progress of the disorder, and sustain the vital powers. A hot bath (102°-106°), in which the patient is to remain a short time only, and to be immediately wrapped in blankets, often gives some relief. When the surface generally or extremities are cold, friction or sinapisms may be used ; blood taken by cups or leeches from the back of the neck, followed by counter-irritation or a blister, appear to be of benefit in many cases. Of all the drugs given internally, Dr. Clymer says that opium has undoubtedly been of the greatest service in subduing the virulence of the symptoms. Quinine, in cases where malaria aggravates the disorder, is also well spoken of. Ether and chloroform inhalations have been used as sedatives; and tincture of cantharides are used in cases marked by extreme depression. The hypodermic injection of morphia about the seat of pain is recommended. The bowels ought to be kept free by purgative enemata, containing croton oil, turpentine, etc. Nutritious and suitable food must be taken, when possible, at short intervals, and through the night as well as the day. During convalescence fresh air, good diet, and tonics are required.

In regard to the extent to which opium has been used in this disease, Dr. Elisha North, who wrote upon this subject in 1811, relates a case of a young woman, about 20 years of age, who recovered from the disorder, being very violently attacked, and a high delirium with great distress supervening, took more than a quart of brandy and not less than twenty grains of good Turkey opium, in less than twelve hours, before any material mitigation of her disorder could be obtained; and there was not the least appearance of intoxication in the case. He also states that he was frequently obliged to give ten grains of opium for a dose in some of the most violent cases, attended with strong spasms, and he never knew it to produce stupor in a single instance.

Bromide of potassium has been given, and as it may be a valuable adjuvant in controlling some of the symptoms, it merits a fair trial, but not to the exclusion of other remedies.

